# The clinical landscape of bladder cancer treatment: a brief report on new developments and future perspectives in pharmaceutical interventions

**DOI:** 10.3389/fonc.2026.1747070

**Published:** 2026-09-18

**Authors:** Xiao Xu, Hai-Yan Hao, Hai-Yan Chen

**Affiliations:** 1 Research Center of Molecular Medicine (Ph.D. Lab), Nantong Health Vocational College, Nantong Health College of Jiangsu Province, Nantong, China; 2 Clinical Research Unit, Affiliated Hospital of Nantong University, Nantong, China; 3 Department of Urology, Nantong Hospital of Traditional Chinese Medicine, Nantong, China

**Keywords:** bladder cancer, clinical landscape, clinical trial, pharmaceutical interventions, urological cancer

## Abstract

This study systematically delineates the clinical research status and trends in bladder cancer (Bca) therapeutics through an analysis of 1024 Bca drug treatment clinical trials identified from a harmonised search of the Trialtrove database, ClinicalTrials.gov, and the World Health Organization International Clinical Trials Registry Platform (WHO ICTRP) database as of December 31, 2025. The results demonstrate that the overall number of clinical trials has tended to increase, with a subsequent decline observed in the post-COVID-19 period; the United States has led research efforts, followed by China, the United Kingdom, Canada, and Australia, which together account for 68.6% of all registered trials; the therapeutic focus has shifted from traditional chemotherapy toward immunotherapy (38.4% of trials, primarily targeting PD-1, PD-L1 and ICIs) and targeted therapies [24.7% of trials, e.g., FGFR inhibitors and Nectin-4-targeting antibody–drug conjugates (ADCs)]. Among validated clinical advances, the EV-302 trial established enfortumab vedotin plus pembrolizumab as the first-line standard of care for locally advanced or metastatic urothelial carcinoma. However, the high trial registration frequency does not uniformly translate into clinical benefit, as exemplified by the subsequent withdrawal of atezolizumab’s bladder cancer indications following negative confirmatory phase III results. In the future, molecular subtype-based personalized treatment strategies, including circulating tumor DNA (ctDNA)-guided adjuvant therapy, novel ADC combination strategies, and artificial intelligence (AI)-driven multiomics integration, will be pivotal for enhancing therapeutic outcomes in Bca.

## 1 Introduction

Bladder cancer (Bca) is a malignant condition that affects the urinary bladder and typically originates from the urothelial cells that line the bladder (1). Bca represents a significant global health challenge characterized by marked epidemiological disparities and an increasing disease burden (2). From a pathological perspective, Bca is classified into nonmuscle-invasive bladder cancer (NMIBC), which accounts for approximately 75% of cases, and muscle-invasive bladder cancer (MIBC), accounting for approximately 25% of cases (3). These subtypes exhibit distinct molecular profiles and clinical outcomes. NMIBC is often characterized by high-frequency TERT promoter mutations and FGFR3-activating mutations, whereas MIBC typically has a higher tumor mutation burden and inactivation of tumor suppressor genes such as TP53 (4).

The impact of Bca on patients is comprehensive and enduring, substantially diminishing their quality of life (5). Concerns about body image and potential odors from urinary diversions can lead to social withdrawal and restricted participation in activities. Psychologically, patients commonly endure considerable emotional distress, including fear and anxiety regarding disease progression, treatment side effects, and an uncertain future (6). Furthermore, the high recurrence rate of bladder cancer, particularly NMIBC, necessitates long-term, close monitoring, perpetuating a state of vigilance and anxiety about potential disease recurrence for survivors (7).

Although Bacillus Calmette-Guérin (BCG) has been the standard treatment for nonmuscle-invasive bladder cancer (NMIBC), the global BCG shortage (since 2013) and the lack of response to BCG therapy in some patients have necessitated the search for alternative treatment methods to improve outcomes for NMIBC patients (8). For example, intravesical gene therapy, such as nadofaragene firadenovec, and the systemic PD-1 inhibitor pembrolizumab are used for the treatment of BCG-unresponsive nonmuscle-invasive bladder cancer (NMIBC).

Moreover, for muscle-invasive and metastatic bladder cancer, despite the clinical application of surgical procedures and perioperative chemotherapy (radical cystectomy combined with cisplatin-based neoadjuvant/adjuvant chemotherapy serves as the cornerstone of management for muscle-invasive bladder cancer), the long-term survival rates of these patients have not improved significantly (4).

For advanced/metastatic bladder cancer, therapeutic options have been expanded to include drugs such as immune checkpoint inhibitors, targeted therapies (the FGFR inhibitor erdafitinib), and antibody–drug conjugates (ADCs) (the Nectin-4-targeting agent enfortumab vedotin), leading the treatment of Bca into the era of immunology/targeted therapy. However, core challenges such as heterogeneous therapeutic efficacy, toxicity management, and the lack of individualized regimens remain to be addressed (9).

The clinical trial landscape of Bca offers a comprehensive perspective, identifying gaps in current treatments, assessing the latest advancements in clinical trial research for Bca, and providing directions for future research endeavors. This study systematically analyzes the clinical trial landscape of Bca therapeutics, identifies critical gaps in current strategies, and evaluates the translational potential of pharmacological therapy on the basis of recent advancements. Our findings can inform the optimization of precision medicine paradigms for Bca pharmacological management and guide future research directions.

## 2 Materials and methods

### 2.1 Data source and search strategy

This comprehensive analysis aimed to systematically map the clinical trial landscape for pharmacological interventions in bladder cancer (Bca). The data were sourced from three databases: the Trialtrove database (owned by Citeline Clinical Intelligence), ClinicalTrials.gov, and the WHO ICTRP. We conducted a systematic query of the database on Dec 31, 2025. The specific search criteria of the Trialtrove database included the following: Drug disease: Bladder cancer; Drug disease group: Oncology; and Medical Subject Headings (MeSH) term IDs D008495 and D001749. To enhance coverage and enable cross-validation, ClinicalTrials.gov and the WHO ICTRP were additionally queried on the same date using a harmonized core set of condition terms tailored to each platform’s syntax. For ClinicalTrials.gov, the condition field was set to: “bladder cancer” OR “urinary bladder neoplasms” OR “bladder carcinoma” OR “urothelial carcinoma” OR “bladder urothelial carcinoma” OR “transitional cell carcinoma of the bladder” OR “vesical carcinoma” OR “muscle invasive bladder cancer” OR “non-muscle invasive bladder cancer”. For the WHO ICTRP, the same core condition terms were used with minor adaptations to comply with the following character limits: “bladder cancer” OR “bladder carcinoma” OR “urothelial carcinoma” OR “bladder urothelial carcinoma” OR “transitional cell carcinoma of the bladder” OR “vesical carcinoma” OR “muscle invasive bladder cancer” OR “nonmuscle invasive bladder cancer”. Intervention type was filtered into drug, biological, and genetic. The complete search strategies for all three databases are provided in **Supplementary Table 1**.

### 2.2 Study selection and eligibility criteria

The inclusion criteria were as follows (1): interventional clinical trials (all phases, I-IV) (2); the enrolled population consisted of human patients with a confirmed diagnosis of bladder cancer; and (3) primary interventions involving pharmacological agents (including small molecules, biologics, antibody–drug conjugates, and cellular therapies). Studies were excluded if they were (1) non-bladder/urothelial cancer as the primary indication (2); observational studies or meta-analyses, diagnostic or biomarker-only studies (2); nonpharmacological interventions such as dietary supplements; or (3) lacked sufficient critical details on key parameters (status, phase, interventions, *etc.*) (4). eligibility confirmed but therapeutic category, phase label, or drug target unassignable.

### 2.3 Data extraction and management

All the extracted data were independently cross-verified by two researchers (XX and HYH). Any discrepancies were resolved through discussion and verified by a third researcher (HYC). Cohen’s kappa coefficient exceeded 0.85 across all the classification dimensions, indicating strong inter-rater reliability. The details of these clinical trials, including the generic drug name, drug name, target, treatment category, summary, latest change, status, phase, drug, disease, initial date, enrollment, interventions, locations, drug country, region, drug country, mechanism of action, development status, therapeutic class, drug type, target family, and drug disease group, are compiled and included in **Supplementary Table 2**.

### 2.4 Data cleaning, handling of duplicates and criteria for trial categorization

#### 2.4.1 Data cleaning

- Drug name standardization: All variant spellings, trade names, development codes, and abbreviated forms referring to the same pharmacological substance were mapped to a single canonical World Health Organization International Nonproprietary Name (WHO INN). For example, “pembrolizumab”, “MK-3475”, and “Keytruda^®^” were mapped to “pembrolizumab”.
- Molecular target harmonization: Synonymous target names were consolidated using UniProt canonical gene identifiers and cross-referenced against the HUGO Gene Nomenclature Committee (HGNC) database. For example, PD-L1/CD274 was unified as a single node.
- Field completeness audit: Before analysis, the completeness of the required analytical fields was evaluated across all the retrieved records: trial status, clinical phase, primary intervention, therapeutic class, drug target, drug disease group, and initial date. Records with simultaneously missing values in all three fields of trial status, clinical phase, and primary intervention were classified as eligibility-unconfirmable and excluded. Records that passed the eligibility assessment but had missing data for therapeutic class, drug target, or initial year such that reliable assignment was impossible were likewise excluded.
- Data currency verification: A random 10% sample was cross-checked against primary ClinicalTrials.gov or ICTRP registration records; discrepancies in status label, phase assignment, or date were corrected to match the primary registry record.

#### 2.4.2 Handling of duplicates

Clinical trial registration numbers (NCT, EudraCT, ISRCTN, ChiCTR, etc.) were used as the primary matching key to identify records referring to the same registered trial across the three source databases (Trialtrove, ClinicalTrials.gov, and the WHO ICTRP). For clinical trials initiated before the implementation of mandatory registration policies (i.e., prior to the ICMJE 2005 policy), a supplementary fuzzy matching approach based on four fields was employed: trial title, primary sponsor name, primary intervention, and study start year. All potential duplicate records thus identified were manually reviewed by two independent researchers (XX and HYH) prior to removal. No records matched solely by fuzzy matching were removed without manual confirmation.

#### 2.4.3 Criteria for trial categorization

- Therapeutic category: The main therapeutic categories (chemotherapy, immunotherapy, targeted therapy, and gene therapy) were assigned on the basis of the “therapeutic class” field in Trialtrove and independently verified by two researchers (XX and HYH) against major pharmacological references. For combination regimens, categorization was based on the primary investigational drug specified in the trial registration record; standard-of-care background agents were not used for the primary classification. In addition, the following prespecified rules were applied: immune checkpoint inhibitors (ICIs) were classified as immunotherapy; antibody-drug conjugates (ADCs) were classified as targeted therapy on the basis of their core mechanism of targeted delivery, with the sole exception of when an ADC in combination with an immune checkpoint inhibitor was employed as the primary investigational strategy and immune synergy was explicitly stated as the primary study objective, the trial was categorized as immunotherapy; CAR-T-cell products were classified as gene therapy according to FDA/EMA regulatory definitions; and intravesical Bacillus Calmette-Guérin (BCG) was classified as immunotherapy.
- Clinical phase: Phase labels were primarily sourced from the “Phase” field in Trialtrove, which aligns with the standardized phase definitions used by ClinicalTrials.gov (based on FDA classification). These labels were further verified and, where discrepancies existed, corrected against primary ClinicalTrials.gov or ICTRP registration records through cross-validation. Records labelled “Early Phase 1” were reclassified as Phase I, as this designation closely overlaps with Phase I in trial design characteristics. Records with the phase label “Not Applicable” were excluded during the eligibility assessment.
- Molecular target standardization: For variant names of the same target (e.g., PD-L1/CD274), the nomenclature was unified using the UniProt canonical gene name cross-referenced against the HGNC database.

## 3 Results

The complete study selection process is shown in **Supplementary Figure 1**. A total of 4,054 records were identified, of which 1,636 duplicates were removed, leaving 2,415 records for screening. Of these, 1,051 were excluded on the basis of predefined criteria, including nondrug interventions, nonbladder/urothelial cancer indications, and other ineligible study types. A total of 1,364 records were retrieved and assessed in full. Following the full-text review, 340 records were excluded, including nonpharmacological studies (n = 142) and nonbladder/urothelial cancer studies (n = 58). Seventeen reports were excluded because of insufficient information to confirm eligibility, as key trial-defining fields (status, phase, and intervention) were all missing. An additional 123 reports met the inclusion criteria but lacked sufficient detail for reliable classification of core analytical variables and were excluded to avoid misclassification bias. In total, 1,024 unique clinical trials were included in the final analysis.

As shown in **Figure 1**, the results revealed an overall increasing trend in the number of clinical trials after 2000, followed by a sharp decline after 2020, possibly associated with the COVID-19 pandemic (**Figure 1**). Notably, the number of clinical trials reached its peak in 2025 (n = 152). Furthermore, the majority of trials were concentrated in Phase I (n = 232; 22.66%) and Phase II (n = 472; 46.09%) (**Figure 1**). The distribution of trial status across phases revealed that phase III trials had the greatest absolute number of completed trials (n = 49; 28.2% of all phase III trials), whereas phase I and phase II trials had the greatest proportions of currently open studies (**Figure 1**). Notably, among all the registered trials, Phase IV trials accounted for only 2.15% (n = 22). Notably, the apparent growth in trial registrations observed in 2005 partially reflects improved registration compliance following the ICMJE mandatory registration policy (2005) rather than purely real increases in research activity.

**Figure 1:**
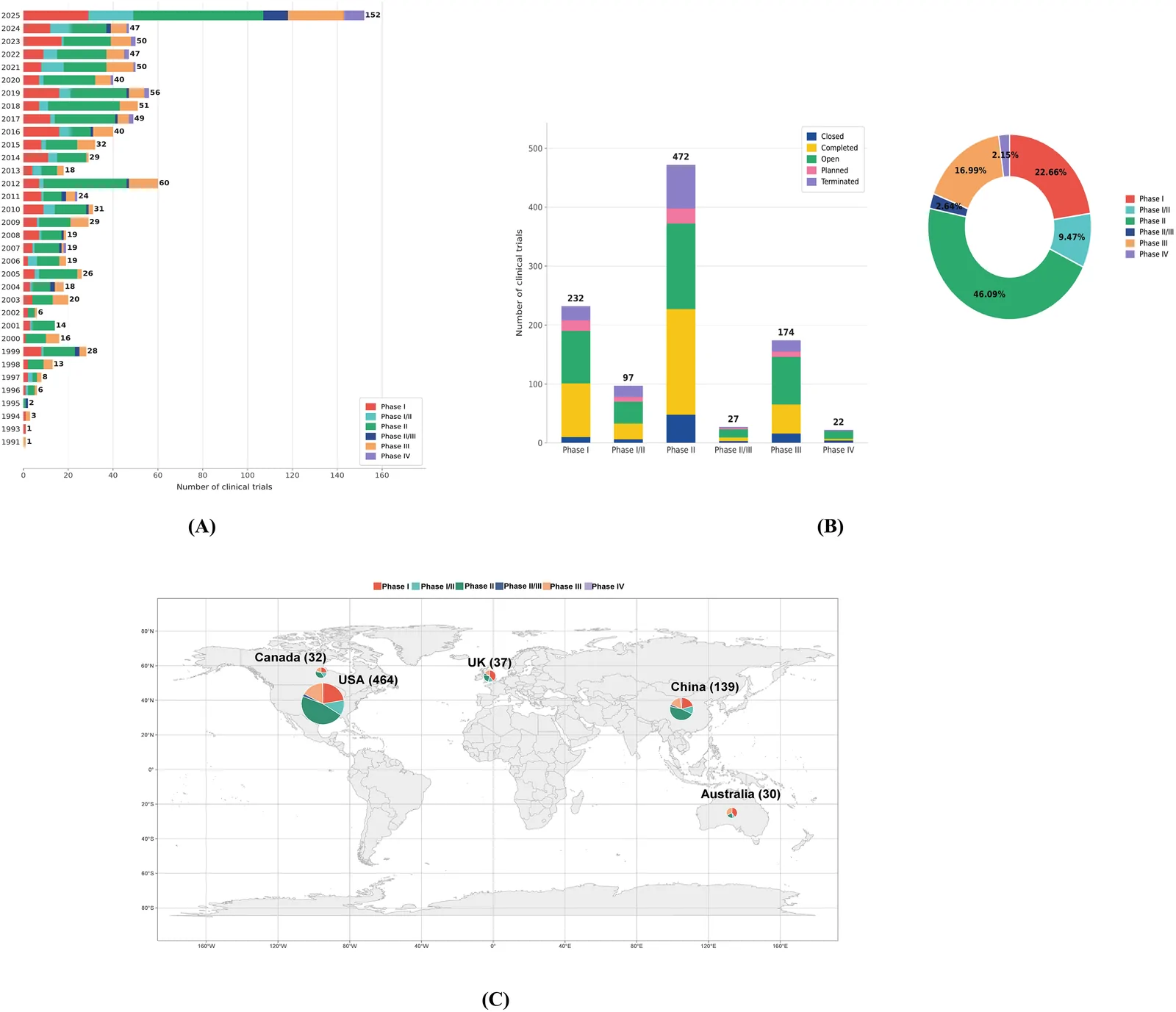
**(A)** The annual number of clinical trials for bladder cancer from 1991 to 2025, stratified by clinical phase (Phase I, Phase I/II, Phase II, Phase II/III, Phase III, and Phase IV). **(B)** Left: The distribution and completion status (Closed, Completed, Open, Planned, Terminated) of clinical trials across Phase I (n = 232), Phase I/II (n = 97), Phase II (n = 472), Phase II/III (n = 27), Phase III (n = 174), and Phase IV (n = 22). Right: Donut chart showing the proportional distribution of all trials by phase. **(C)** World map showing the top 5 countries by number of Bca drug clinical trial sites: the United States (n = 464), China (n = 139), the United Kingdom (n = 37), Canada (n = 32), and Australia (n = 30). Proportional pie charts at each country location indicate the phase distribution of trials (red: phase I; cyan: phase I/II; green: phase II; dark blue: phase II/III; orange: phase III; purple: phase IV).

The five countries with the most Bca drug clinical trials are highlighted in **Figure 1**. These are located in North America (the United States [n = 464] and Canada [n = 32]), Europe (the United Kingdom [n = 37]), East Asia (China [n = 139]), and Oceania (Australia [n = 30]). Together, these five countries account for 68.6% of all registered trials (n = 702 of 1,024). By far, the United States has the greatest number of clinical drug trials. Notably, the combination of tislelizumab with BCG has entered phase IV clinical trials at Shanghai Jiao Tong University in China (NCT05580354), which assess long-term efficacy and safety postmarketing. However, as the study was conducted exclusively in China, caution is needed when interpreting the data regarding the use of the drug.

Clinical trials of immunotherapy and targeted therapy constituted the largest treatment categories (**Figure 2**). Among these, immunotherapy trials accounted for the greatest proportion (n = 393; 38.4%), followed by chemotherapy (n = 343; 33.5%), targeted therapy (n = 253; 24.7%), and gene therapy (n = 35; 3.4%) (**Figure 2**). The phase distribution across categories reflects the maturity of each therapeutic modality: immunotherapy and targeted therapy are concentrated in phases I and II, indicating that these emerging modalities remain in active early-stage development; in contrast, chemotherapy accounts for a relatively higher proportion of phase III trials, reflecting a more mature clinical development pipeline (**Figure 2** and **Supplementary Table 3A**). Immunotherapy had the highest proportion of open trials (46.1%), substantially exceeding the 25.4% observed for chemotherapy, indicating that immunotherapy remains the most intensively pursued area in current clinical research. Chemotherapy accounted for the greatest proportion of completed trials (45.5%), which is consistent with its established role as a standard of care and the accumulated history of clinical investigation. Notably, the proportions of terminated trials for gene therapy and immunotherapy were 17.1% and 12.0%, respectively, suggesting that these rapidly evolving fields are accompanied by a relatively high risk of developmental failure or early termination (**Supplementary Table 3B**). The chord diagram in **Figure 2** illustrates the co-occurrence patterns among the different treatment categories. The strongest combinatorial associations were observed between chemotherapy and immunotherapy (n = 46 cooccurring trials) and between immunotherapy and targeted therapy (n = 15 cooccurring trials), suggesting that combination strategies have become a clear clinical development trend. The current drug development pipeline for bladder cancer therapy is shown in **Supplementary Figure 2**.

**Figure 2:**
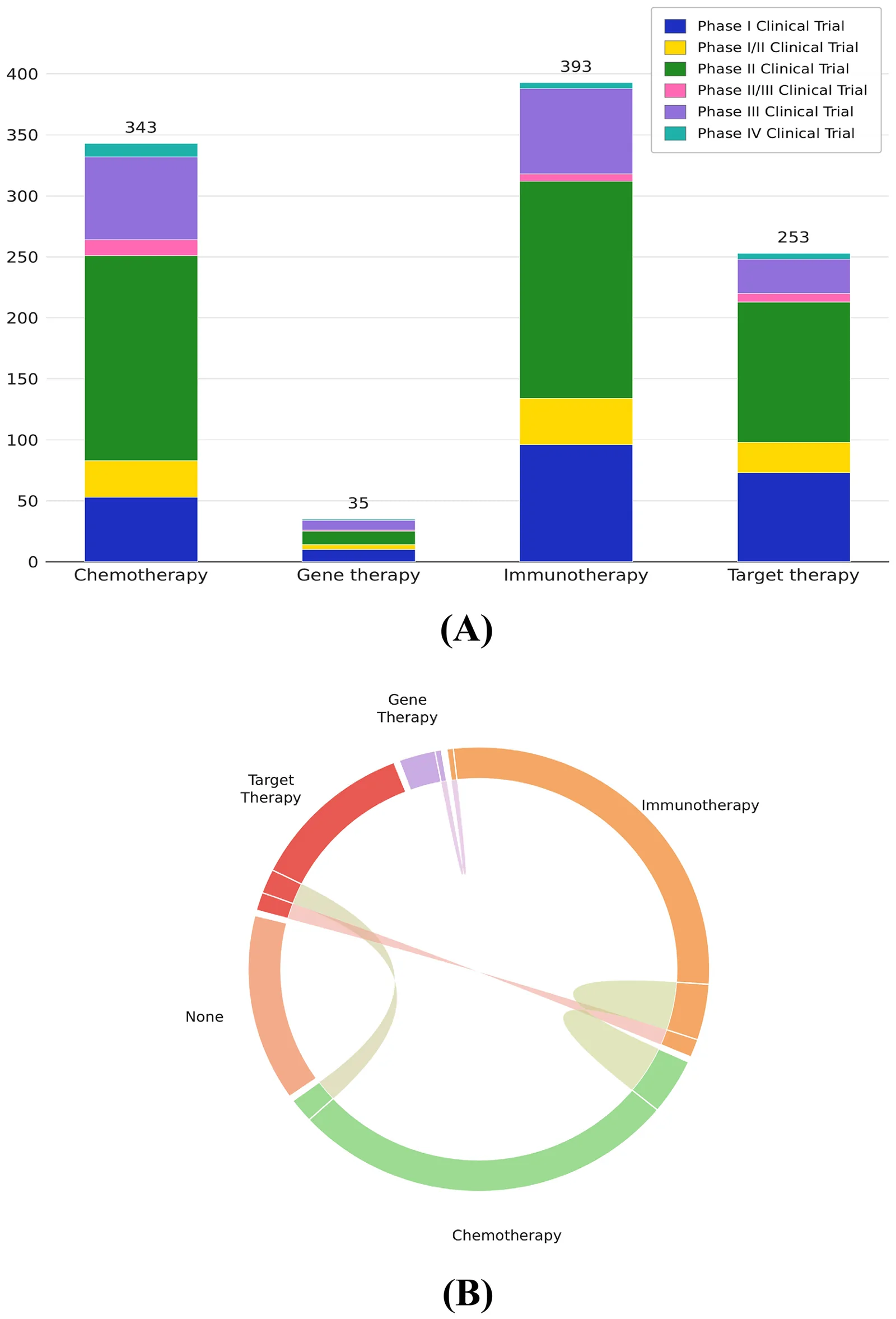
**(A)** Stacked bar chart showing the clinical phase distribution of trials within each primary treatment category: chemotherapy (n = 343), gene therapy (n = 35), immunotherapy (n = 393), and targeted therapy (n = 253). **(B)** Chord diagram illustrating co-occurrence patterns among treatment categories across all trials.

For example, cisplatin-based chemotherapy is the standard treatment for metastatic bladder cancer, and PD-1/PD-L1 inhibitors have shown promising effects. Additionally, combination therapy with pembrolizumab (an immune checkpoint inhibitor) and enfortumab vedotin (an antibody–drug conjugate-targeting agent) has been granted Breakthrough Therapy Designation by the FDA (10).

In the Sankey diagram (**Supplementary Figure S3**), the width of the flow lines between nodes quantifies the distribution characteristics of the clinical usage frequency of drugs. Among immune checkpoint inhibitors, PD-L1 antagonists (including atezolizumab, durvalumab, and avelumab) and PD-1 antagonists (including pembrolizumab and nivolumab) dominate the flow, together accounting for the majority of immune checkpoint inhibitor trials. This may be related to the wide range of indications for PD-L1/PD-1 inhibitors in advanced or metastatic bladder cancer (4). As the most commonly used ADC drug, enfortumab vedotin has a medium flow line width, reflecting that ADC drugs, as emerging therapeutic approaches, have demonstrated certain application value in patients with prior treatment failure, but their usage frequency is still lower than that of immune checkpoint inhibitors. The flow line widths of docetaxel and gemcitabine are maintained at a medium level, indicating that traditional chemotherapeutic drugs still remain stable in the treatment of bladder cancer. These agents target the fibroblast growth factor receptor (FGFR) family, and the differences in flow line widths among erdafitinib, infigratinib, and futibatinib are small. These findings suggest that in bladder cancer subtypes with abnormal FGFR expression, the application frequency of such targeted drugs is relatively uniform, reflecting precision in the treatment of specific molecular subtypes. Cretostimogene grenadenorepvec, a gene therapy agent, has the narrowest flow line width, suggesting that it is still in the early stage of clinical research and that gene therapy has limited practical application in bladder cancer treatment.

The associations among bladder cancer therapeutic drugs, mechanisms of action, and molecular targets were further systematically displayed through a network diagram (**Figure 3**). Immune checkpoint inhibitors, the largest functional category (n = 214), use PD-1, CTLA4, and CD274 (PD-L1) as core targets, with nodes showing dense connections and a radial distribution. Antibody-drug conjugates (ADCs) mainly target Nectin-4, with independent nodes and clear connecting lines, reflecting the precision of the use of ADC drugs (such as enfortumab vedotin) in targeted therapy. The mechanism of action involves mainly cytotoxicity through antibody-mediated processes, with few cross-interactions with other types of drugs. Immuno-oncological therapies cover targets such as toll-like receptors, and the nodes are relatively scattered, suggesting that this category of drugs may function by activating innate or adaptive immune pathways. DNA inhibitors target molecules related to DNA damage repair, such as CHK1. The nodes are concentrated with tight connections, indicating that traditional chemotherapeutic drugs exert their cytotoxic effects by interfering with DNA replication or repair.

**Figure 3:**
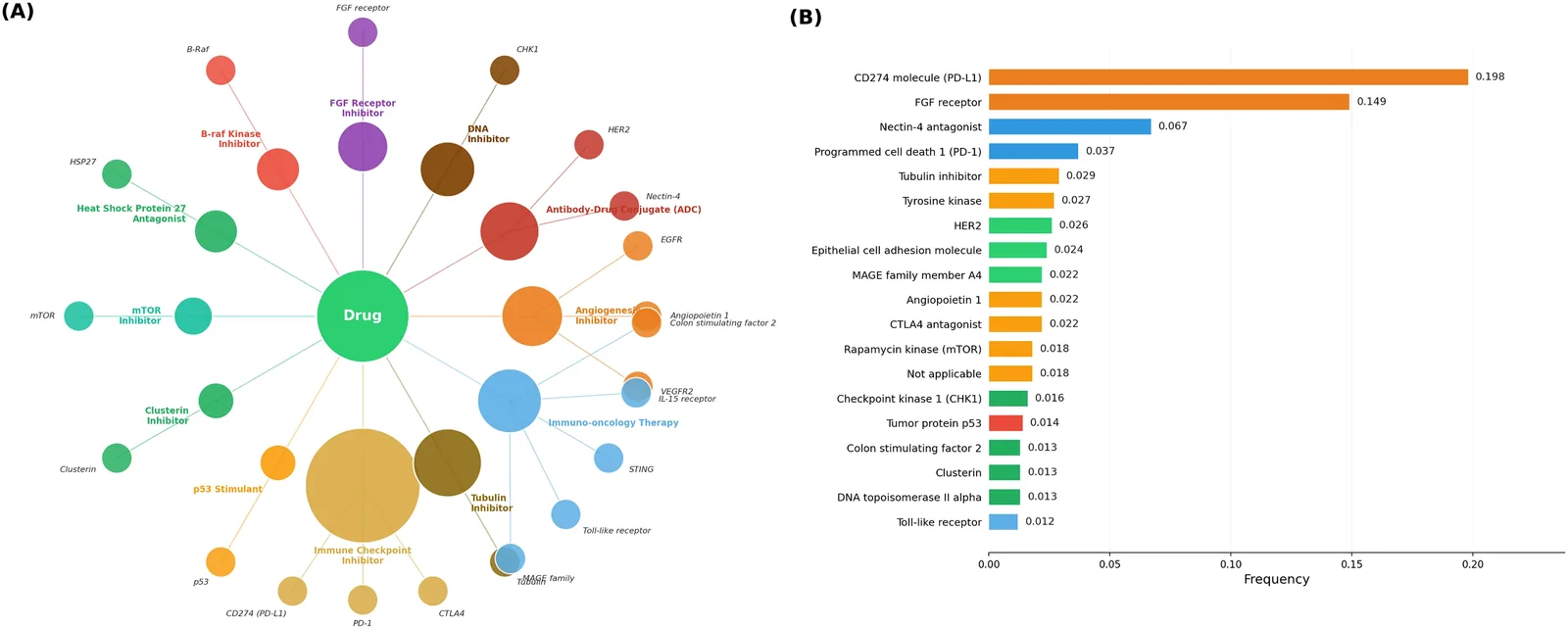
**(A)** Network diagram illustrating the associations among bladder cancer drugs (central node), functional drug categories (inner nodes, sized by trial frequency), and individual molecular targets (outer nodes). **(B)** Horizontal bar chart showing the relative frequency of the top therapeutic targets across all 1,024 trials.

To quantify the application frequency of each target in bladder cancer treatment, this study conducted frequency statistics on drug–target associations (**Figure 3**). The results revealed that CD274 (PD-L1), FGF receptors, and Nectin-4 antagonists are high-frequency targets, with frequency values of 0.198, 0.149, and 0.067, respectively, which are significantly greater than those of other targets. These findings suggest that PD-L1 inhibitors, FGFR-targeted drugs, and Nectin-4 ADC drugs are the most widely used drugs in clinical practice, which is highly consistent with the current research hotspots and approved indications in bladder cancer treatment. In particular, immune checkpoint inhibitors targeting the CD274 molecule have shown durable responses in patients with advanced metastatic urothelial carcinoma and have become the first-line treatment for cisplatin-ineligible patients. FGFR3 is a commonly mutated gene in bladder cancer, and the pan-FGFR inhibitor erdafitinib has been approved by the FDA for second-line treatment regimens (11). Tyrosine kinases, tubulin, and HER2 are medium-frequency targets with frequency values ranging from 0.026 to 0.029, indicating that such drugs have certain application value in specific subtypes or refractory cases but have not yet become mainstream treatment options. mTOR, CHK1, Clusterin, etc., are rare targets with frequency values less than 0.025, suggesting that they may be in the clinical exploration stage or only applicable to highly specific molecular subtypes.

Changes in the research and development (R&D) cycle of bladder cancer drugs over time are shown in **Figure 4**. Trials initiated before 2015 and with long durations (≥10 years) had average durations ranging from approximately 10 to 25 years, reflecting the historically lengthy timelines of classical drug development. In contrast, from 2015 onwards, trials predominantly fall into the short-duration category (<10 years), with average durations declining progressively to less than 8 years by recent years. This shortening trend may be attributed to multiple factors, such as innovations in scientific research technologies, optimization of research methods, and improvements in clinical trial procedures.

**Figure 4:**
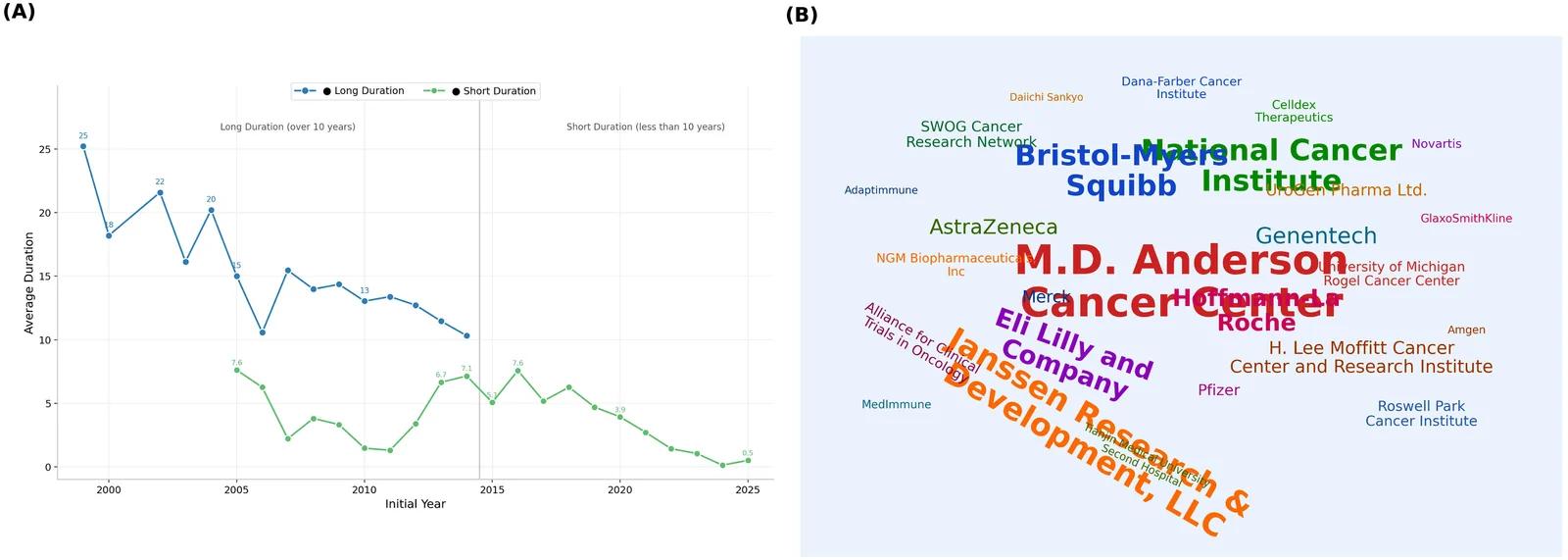
**(A)** Line chart depicting the average trial duration (in years) by trial initiation year, stratified into long-duration trials (≥10 years, blue) and short-duration trials (<10 years, green). **(B)** Word cloud in which font size is proportional to the number of sponsored trials per institution.

The funding sources for bladder cancer drug research and development are presented in **Figure 4**. M.D. Anderson Cancer Center leads all sponsors with 85 associated trials, followed by Janssen Research & Development, LLC (n = 62), the National Cancer Institute (n = 57), and Bristol-Myers Squibb (n = 55), indicating that both leading academic cancer centers and major pharmaceutical companies play pivotal roles in driving Bca drug development. Several research consortia and networks, such as the Alliance for Clinical Trials in Oncology and the SWOG Cancer Research Network, also play important roles in bladder cancer drug research and development.

## 4 Discussion

On the basis of an analysis of the global bladder cancer clinical trial landscape, this study maps the research investment landscape across 1,024 registered trials. The observed increase in trial registration activity directed toward immunotherapy, ADC combinations, and targeted therapy directly reflects evolving research priorities and investment patterns. However, shifts in trial volume are process metrics and should not be conflated with evidence of actual changes in clinical practice; establishing clinical impact requires outcome data rather than trial counts alone. To clarify this distinction, the following discussion integrates key phase-III outcome evidence to identify where the trial volume trends we observe have been validated by clinical results and where high trial activity has led to negative outcomes.

BCG remains the first-line treatment for high-risk nonmuscle-invasive bladder cancer (NMIBC); however, patients face persistent supply constraints, stemming primarily from a highly concentrated supply chain, complex manufacturing challenges, and a systemic lack of incentives. For example, Merck, as the sole supplier of intravesical BCG in the U.S. market, has limited production capacity (12, 13). Furthermore, BCG, a live bacterial preparation, requires a production process of approximately three months and is susceptible to interruptions due to contamination (12). Concurrently, the issue of BCG unresponsiveness has become a significant clinical challenge that urologists must consider in the management of Bca. From a mechanistic perspective, BCG treatment failure in high-risk nonmuscle-invasive bladder cancer (NMIBC) is closely linked to tumor-intrinsic immune evasion involving IDO1-mediated immunosuppression. On the basis of a discovery cohort of 73 BCG-naïve T1 high-risk NMIBC patients (responders and nonresponders; dataset GSE154261), Turdo et al. (14) derived a gene expression signature and identified indoleamine 2,3-dioxygenase 1 (IDO1) as a key determinant of BCG treatment outcome. These findings were subsequently validated in an independent cohort of 75 patients using pretreatment formalin-fixed paraffin-embedded (FFPE) specimens, where elevated IDO1 expression was consistently associated with BCG failure (14). Pathway analysis revealed that IDO1 transcription is regulated by STAT1 signalling. Coexpression data demonstrated that the expression of IDO1 strongly correlated with that of immune checkpoints, including PD-1, PD-L1, PD-L2, LAG3, and CTLA4, suggesting that these genes may participate in an immunosuppressive program involving T-cell dysfunction (14). The requirement of intact immune activation for BCG efficacy is well established. In this study, tumor immune microenvironment analysis revealed that responder patients exhibited increased numbers of tumor-reactive B cells, CD4^+^ T cells, CD8^+^ T cells, and dendritic cells; in contrast, nonresponder patients uniquely displayed the presence of CD4^+^ regulatory T cells (Tregs), which suppressed tumor-specific CD8^+^ T-cell cytotoxicity. Moreover, responders demonstrated more pronounced infiltration of cytotoxic CD8^+^/granzyme B^+^ T cells, whereas such cells were scarce in nonresponders and were confined mainly to stromal regions (14). These observations indicate that the tumor microenvironment of IDO1-high tumors is unfavourable for BCG-mediated immunotherapy, thereby counteracting the immune priming essential for tumor clearance. On the basis of this evidence, BCG unresponsiveness is not merely a feature of “high-risk” disease stage but rather a consequence of a specific immunosuppressive programme. Turdo et al. (14) reported that IDO1 promotes epithelial–mesenchymal transition (EMT) in bladder cancer via the IL-6/STAT3/PD-L1 signalling pathway and that various IDO1 inhibitors are currently under clinical evaluation, with combination strategies involving other immunotherapies demonstrating synergistic potential (14). Importantly, the IDO1 finding may further extend to biomarker application: IDO1 protein expression in pretreatment FFPE specimens can be assessed by immunohistochemistry, offering a practical tool for stratifying high-risk NMIBC patients before BCG initiation.

To address the BCG dilemma, in addition to pembrolizumab and nadofaragene, numerous novel intravesical agents, such as Vicineum and ALT-803, as well as sequential combination chemotherapy regimens (e.g., gemcitabine plus docetaxel), have demonstrated potential in BCG-unresponsive patients (13, 15, 16). The KEYNOTE-057 trial (17) reported a complete response rate of 41% with pembrolizumab; however, only approximately 19% of responders maintained the response at 12 months, underscoring the challenge of achieving durable efficacy and highlighting the urgent need for developing subsequent therapies. For patients who are either unwilling or unable to undergo cystectomy, these emerging options represent valuable bladder-preserving opportunities.

Among the registered trial trends, the growth of chemotherapy-immunotherapy combination studies (**Figure 2**; n = 46 cooccurring trials) is the trend most strongly validated by phase III outcome data. The CheckMate 901 trial (NCT03036098) (18) demonstrated that compared with chemotherapy alone, nivolumab plus gemcitabine-cisplatin significantly improved OS in patients with cisplatin-eligible metastatic urothelial carcinoma (median OS 21.7 vs. 18.9 months; HR = 0.78; *P* = 0.02).

The co-occurrence of immunotherapy-targeted therapy (n = 15; **Figure 2**) was validated by the EV-302 trial (19). The EV-302 trial (NCT04223856) demonstrated that enfortumab vedotin (a Nectin-4-targeting ADC, classified as targeted therapy) plus pembrolizumab (a PD-1 inhibitor, classified as immunotherapy) nearly doubled the median overall survival compared with platinum-based chemotherapy in previously untreated patients with locally advanced or metastatic urothelial carcinoma (31.5 vs. 16.1 months; HR = 0.47; 95% CI 0.38-0.58; *P* < 0.001). On the basis of these results, the FDA granted regular approval on December 2023, and the European Commission granted marketing authorization on August 2024 (European Commission, 2024), establishing EVs plus pembrolizumab as the current international first-line standard of care for locally advanced or metastatic urothelial carcinoma (19).

Beyond this landmark validation, additional ADC-ICI combinations have shown encouraging early-phase signals that are consistent with the trend but have not yet met the same evidentiary standard. The combination of RC48-ADC (disitamab vedotin) with PD-1 inhibitors has demonstrated notable efficacy in bladder-preserving therapy for muscle-invasive bladder cancer (MIBC), achieving a pathological complete response rate of 82.35% compared with 47.62% for the GC regimen plus immunotherapy, with comparable safety profiles (20). In a study of locally advanced or metastatic urothelial carcinoma, RC48-ADC alone or combined with PD-1 inhibitors yielded an objective response rate of 63.2% and a 12-month overall survival rate of 76.7% among 38 patients (21). These findings are promising and align with the broader momentum toward ADC-ICI combinations; however, they currently lack confirmation from phase III trials with OS endpoints.

However, the number of registered clinical trials does not necessarily directly reflect the validity of clinical efficacy; rather, it more often represents research investment and development priorities, and the relationship between such investment and ultimate clinical benefit is not simple linear.

Atezolizumab (anti-PD-L1) was the most frequently registered drug in our dataset (**Supplementary Figure S3**, n = 69 trials), yet both of its FDA bladder cancer indications were ultimately withdrawn. The second-line indication was voluntarily withdrawn in 2021 following the negative confirmatory results of IMvigor211, which failed to meet its primary OS endpoint (HR = 0.87; *P* = 0.41) (22). The first-line indication was subsequently withdrawn in 2022 after IMvigor130 failed to demonstrate a statistically significant improvement in OS. In contrast, the FGFR inhibitor erdafitinib represents a partially validated targeted therapeutic signal. In the phase III THOR trial (NCT03390504) (23), erdafitinib significantly improved OS compared with chemotherapy in patients with FGFR-altered metastatic urothelial carcinoma (median 12.1 vs. 7.8 months; HR = 0.64; *P* = 0.005). However, THOR Cohort 2 demonstrated no significant difference in OS between patients treated with erdafitinib and those treated with pembrolizumab in anti-PD-(L)1-naïve FGFR-altered patients (median 10.9 vs. 11.1 months; HR = 1.18; *P* = 0.18), suggesting that FGFR inhibition does not consistently outperform immunotherapy even in biomarker-selected populations. Collectively, these findings indicate that high trial registration frequency reflects substantial research investment and biological interest but does not necessarily translate into broad or uniformly validated clinical benefit.

The atezolizumab case also illustrates how regional regulatory frameworks shape the relationship between trial volume and clinical practice. The FDA accelerated approval pathway enables market access on the basis of surrogate endpoints such as the objective response rate while requiring postmarketing confirmatory evidence to verify clinical benefit. Accordingly, atezolizumab entered clinical use in 2016 on the basis of the ORR data but was later withdrawn in 2021–2022 after the results of the confirmatory phase III studies failed to demonstrate a significant improvement in OS. The EMA Conditional Marketing Authorization pathway operates under a broadly similar framework, although evidentiary expectations and postmarketing requirements may differ from those of the FDA. Following China’s accession to the ICH in 2017, the NMPA implemented comparable expedited regulatory pathways; however, historical analyses have suggested that only approximately 34.9% of FDA-approved oncology indications (2001–2020) subsequently received corresponding NMPA approval, reflecting differences in regulatory priorities, local data requirements, and healthcare system considerations (24). Therefore, the geographic distribution of registered trials (US [n = 464]; China [n = 139]) should be interpreted as reflecting research investment within distinct regulatory ecosystems rather than equivalent probabilities of approval or treatment adoption.

Mechanistically, this synergistic effect originates from the ability of ADC drugs to precisely deliver cytotoxic payloads, inducing immunogenic cell death (ICD) in tumor cells.​ Preclinical studies have demonstrated that enfortumab vedotin (EV) can induce immunogenic cell death (ICD), a process mediated by its cytotoxic payload, monomethyl auristatin E (MMAE). Mechanistically, MMAE-mediated microtubule disruption triggers concomitant endoplasmic reticulum (ER) stress, leading to the release of key damage-associated molecular patterns (DAMPs), including extracellular ATP secretion and HMGB1 release. These events activate antigen-presenting cells and promote the cross-presentation of tumor antigens to cytotoxic T cells (25). This process exposes tumor neoantigens and remodels the tumor microenvironment (TME), effectively reversing them from an immunosuppressive state to an immunostimulatory state and thereby significantly increasing the efficacy of immune checkpoint inhibitors. The frontier of this combination strategy is now extending beyond advanced disease into the perioperative setting. For example, neoadjuvant immunotherapy, such as pembrolizumab monotherapy in the PURE-01 study (26), achieved a pathological complete response (pCR) rate of approximately 42% in muscle-invasive bladder cancer (MIBC), representing a promising therapeutic signal. Updated 5-year PURE-01 results further reported encouraging long-term outcomes, including a 5-year overall survival rate of 77% (27). However, as PURE-01 was a single-arm phase II study, these findings do not yet establish a new standard of care, and confirmation in randomized phase III trials with survival-based endpoints remains necessary.

However, some Bca patients do not respond well to current standard treatment strategies, and existing treatment options do not meet their needs. Future interventions for Bca should emphasize personalization and explore new therapeutic avenues. Patient stratification on the basis of molecular subtypes has demonstrated substantial clinical value. For example, tumors harboring alterations in the FGFR2 or FGFR3 genes often respond favourably to selective FGFR inhibitors such as erdafitinib, establishing this agent as an important option for pretreated metastatic patients. This paradigm of subtype-guided therapeutic decision-making represents a core tenet of precision medicine. Furthermore, serial ctDNA monitoring is now clinically validated in bladder cancer: the IMvigor011 phase 3 trial demonstrated that ctDNA positivity after radical cystectomy can be used to identify patients who benefit from the adjuvant atezolizumab (hazard ratio for the first event of disease recurrence or death, 0.64; 95% CI 0.47-0.87; *P* = 0.005; hazard ratio for death, 0.59; 95% CI 0.39-0.90; *P* = 0.01), establishing that ctDNA-guided decision-making is a key tool for personalized postsurgical care (28).

Furthermore, the development of novel therapies is being increasingly directed by these precise molecular insights. For example, the efficacy of the Nectin-4-targeting antibody–drug conjugate enfortumab vedotin is closely linked to target expression levels, and its immunomodulatory effects through the induction of immunogenic cell death (ICD) provide a rationale for combination strategies with immune checkpoint inhibitors, highlighting a targeted and mechanism-driven approach to treatment innovation.

Gene therapy, including CAR-T-cell approaches, represented 3.4% of the trials in our dataset (n = 35) (**Figure 2**). However, this proportion should be interpreted cautiously in the context of currently available clinical evidence. CAR-T-cell therapy products, such as CARVYKTI^®^, have achieved remarkable results in patients with hematological malignancies, with an overall response rate (ORR) of 98% (29). Nevertheless, these successes remain limited to blood cancers, and no CAR-T product has yet received regulatory approval for any solid tumor, including bladder cancer. The only registered phase I/II CAR-T clinical trial specifically targeting metastatic urothelial bladder cancer (NCT03185468) has been ongoing for several years and has not published efficacy results, indicating that CAR-T-cell therapy for bladder cancer remains at an early investigational stage rather than representing an established therapeutic advance.

Preclinical studies nevertheless provide proof-of-concept support for continued exploration. Ding et al. (30) demonstrated that CAR-T cells targeting the bladder cancer-specific antigen SIA-IgG exhibited potent antitumour activity in preclinical models. More notably, multiomics analyses within the same study suggested that combining CAR-T-cell therapy with histone deacetylase inhibitors (HDACis), such as vorinostat, may increase CAR-T-cell persistence and partially overcome T-cell exhaustion, which is a major obstacle in solid tumors. These findings highlight potential future optimization strategies but remain preclinical and require substantial clinical validation.

Similarly, recent reports describing the use of donor-derived CAR-T-cell therapy in autoimmune diseases (31) suggest that allogeneic manufacturing approaches may reduce production barriers and improve accessibility to cellular therapies (32). However, these observations are derived from nononcological settings and should not be interpreted as evidence of clinical efficacy in bladder cancer. Overall, the current frequency of gene therapy trial registration in our dataset appears to reflect exploratory research investment and technological interest rather than imminent clinical practice change. Future precision oncology strategies in bladder cancer will likely depend on the integration of multiomics profiling, longitudinal liquid biopsy monitoring, and artificial intelligence-assisted therapeutic selection to enable more individualized treatment sequencing.

## 5 Future perspective

Several priority areas identified by our trial landscape analysis have already advanced to clinical investigation. First, the IMvigor011 trial (NCT04660344) revealed that serial ctDNA positivity after radical cystectomy was associated with a significantly reduced risk of disease recurrence or death (HR 0.64; 95% CI 0.47-0.87; *P* = 0.005) and a significant improvement in overall survival (HR 0.59; 95% CI 0.39-0.90; *P* = 0.01) (28). This paradigm shift establishes ctDNA monitoring as a clinically validated tool for personalized postsurgical care in bladder cancer and is expected to catalyse further registrational studies. Second, personalized cancer vaccines combined with established immunotherapies have emerged as a trial-registered strategy in bladder cancer. The INTerpath-011 trial (NCT06833073) is evaluating the mRNA neoantigen vaccine V940 plus intravesical BCG in high-risk NMIBC, extending the neoantigen vaccine approach, previously demonstrated in melanoma, to bladder cancer. Third, FGFR3-targeted therapies in molecularly selected populations are advancing across multiple clinical trial phases (erdafitinib intravesical delivery, phase 1 [NCT05567185] and vepugratinib, phase 3 [NCT07218380]), reflecting the maturation of the precise FGFR axis identified in our target frequency analysis (**Figure 3**). Fourth, bladder cancer-specific AI applications are already demonstrating clinical utility: an interpretable multimodal deep learning framework predicts pathological complete response to neoadjuvant chemotherapy in MIBC, facilitating personalized treatment optimization (33).

In contrast, several other directions that have attracted considerable scientific interest remain at a predominantly preclinical or early experimental stage in bladder cancer. First, artificial intelligence (AI) and digital health technologies are advancing across multiple fronts simultaneously (34, 35), with emerging applications that include AI-assisted clinical trial design optimization and large language model (LLM)-powered clinical decision support integrated with wearable sensor data. The integration of smart health monitoring and wearable technologies further enables continuous, real-time tracking of patient functional status, treatment-related adverse events, and adherence parameters, thereby extending personalized monitoring beyond clinic visits (36, 37). Although these digital health strategies have not yet been evaluated in formal phase 3 interventional trials in bladder cancer, several feasibility studies and implementation science initiatives are already underway. Collectively, these developments suggest that AI and wearable technology are poised to bridge the gap between the exploratory and clinical tiers of the translational pipeline in bladder cancer. Second, the convergence of single-cell technologies, multiomics, and advanced *in vitro* modelling platforms is generating transformative preclinical insights, yet their translation into bladder cancer clinical practice remains at an early stage (38). Third, plant-derived nanovesicles (PDNVs) represent a conceptually novel nanomedicine platform characterized by low immunogenicity, inherent antitumour bioactivity, and dual therapeutic and delivery functionality (39–42). Importantly, a recent review specifically examined the translational potential of PDNVs in genitourinary tumors and reported that their ability to modulate immune function and reverse chemoresistance offers a promising alternative for overcoming BCG treatment resistance (43). Nevertheless, it must be emphasized that, to date, the PDNV evidence base in bladder cancer has been derived entirely from a retrospective analysis of *in vitro* cell line experiments; no PDNV-based therapeutic has entered clinical trials for any genitourinary malignancy.

## 6 Limitations

Although the Trialtrove database integrates data from tens of thousands of sources and is updated more frequently than traditional government clinical trial registries do, it may still suffer from inherent incompleteness. This could introduce selection bias and limit the comprehensiveness of our analytical results. Consequently, the generalizability of the findings, which are based on a limited number of trials, requires further validation with more extensive evidence.

First, mandatory clinical trial registration was not universally enforced before the ICMJE policy (2005); pre-2005 trials are systematically underrepresented in all registry-based datasets. The growth in registered trial activity observed after 2005 in **Figure 1A** partly reflects increasing registration compliance rather than purely real research growth, and this period should be interpreted with particular caution (44). Second, because Trialtrove predominantly aggregates English-language data sources, it systematically underestimates clinical trial activity for bladder cancer drugs in Asian and low-income country regions. Third, although Trialtrove is updated more frequently than most official registries are, a time lag remains between real-world trial status changes (e.g., from “active” to “completed” or “terminated”) and their reflection in the database, which may affect status-based completion-rate estimates. The 10% random-sample manual cross-verification partially mitigated this concern but could not fully eliminate the error introduced by such update lag. Fourth, the “therapeutic class” field in Trialtrove relies on Citeline’s proprietary classification system, which may show minor inconsistencies with international standard pharmacological classifications such as the WHO ATC system. To address this, all therapeutic categories were independently verified by two researchers (κ > 0.85) following pre-specified decision rules. The assignment of the major categories is consistent across all mainstream classification systems (e.g., immune checkpoint inhibitors were classified as immunotherapy, and antibody–drug conjugates (ADCs) were classified as targeted therapy on the basis of their core mechanism of targeted delivery). This bias therefore has a limited impact on the main conclusions, although a residual risk of category misclassification cannot be entirely excluded. Fifth, this analysis relied exclusively on trial registration data and did not incorporate trial outcome data. Consequently, the clinical value of a specific therapeutic modality cannot be inferred from clinical trial counts alone. Furthermore, the primary endpoints in current clinical trials for bladder cancer drugs predominantly focus on objective efficacy measures, often overlooking patient-reported outcomes (PROs), such as improvements in subjective symptoms such as fatigue, depression, and anxiety. This disconnect may lead to a misalignment between the amelioration of objective indicators and the enhancement of patients’ subjective experiences during clinical treatment, resulting in a failure to fully reflect the true impact of the therapy on quality of life.

## 7 Conclusion

This study analyzes global clinical trials of Bca therapeutics, outlining the current research and development landscape. The results indicate that the clinical research in the United States has led; the treatment paradigm is shifting from traditional chemotherapy to immunotherapies (particularly PD-1/PD-L1 inhibitors) and targeted agents (including FGFR inhibitors and Nectin-4-targeting ADCs), and combination strategies (e.g., immune checkpoint inhibitors + ADC/chemotherapy) demonstrate considerable potential and have achieved breakthroughs in therapeutic design. Importantly, this analysis demonstrated that trial registration volume reflects research investment rather than validated clinical benefit: the most frequently registered agent (atezolizumab) had both FDA indications withdrawn, whereas pivotal combination strategies such as enfortumab vedotin plus pembrolizumab have established new standards of care. Molecular biomarkers, including IDO1 overexpression as a predictor of BCG failure and ctDNA-guided adjuvant therapy validated in IMvigor011, are now transitioning from discovery to practice-guiding tools. Future bladder cancer management will advance along two tracks: the near-term integration of strategies already supported by phase 2–3 evidence (ctDNA-directed therapy, perioperative ADC-ICI combinations, and FGFR-targeted precision therapy), and the longer-term translation of experimental approaches including AI-assisted clinical decision-making and novel drug delivery platforms.

## Data Availability

The original contributions presented in the study are included in the article and its Supplementary Material. The clinical trial records analysed were retrieved from ClinicalTrials.gov (https://clinicaltrials.gov/) and the WHO International Clinical Trials Registry Platform (https://trialsearch.who.int/), both of which are publicly accessible without restriction, and from Trialtrove (Citeline Clinical Intelligence, https://www.citeline.com/), a commercial subscription-based database. The complete search strategies are provided in **Supplementary Table 1**, the record-level dataset of the 1,024 included trials in **Supplementary Table 2**, and the aggregated distributions by clinical phase and trial status in **Supplementary Table 3**.

## Supplementary material

Supplementary Figure 1 Flow diagram.

Supplementary Figure 3 Sankey diagram: drug name, target of action, and drug class.
