# Supplementary material for: The clinical landscape of bladder cancer treatment: a brief report on new developments and future perspectives in pharmaceutical interventions
Source: Front Oncol. 2026 Sep 18;16:1747070. doi: 10.3389/fonc.2026.1747070 (PMC13631784; doi:10.3389/fonc.2026.1747070)
Supplement: Supplementary file 1 [file DataSheet1.pdf]

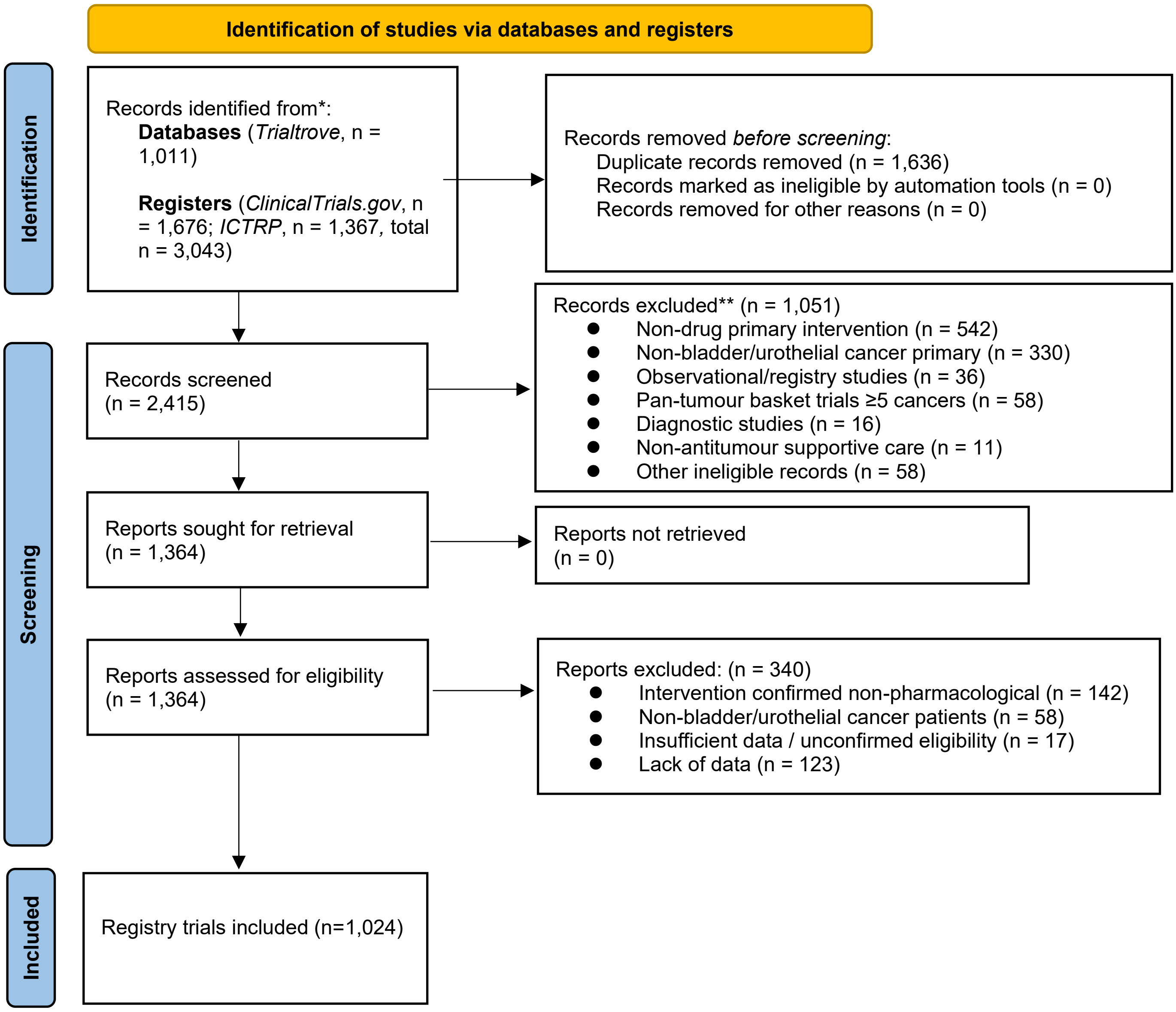
Identification of studies via databases and registers
Identification
Records identified from*:
Databases (Trialtrove, n = 1,011)
Registers (ClinicalTrials.gov, n = 1,676; ICTRP, n = 1,367, total n = 3,043)
Records removed before screening:
Duplicate records removed (n = 1,636)
Records marked as ineligible by automation tools (n = 0)
Records removed for other reasons (n = 0)
Screening
Records screened
(n = 2,415)
Records excluded** (n = 1,051)
Non-drug primary intervention (n = 542)
Non-bladder/urothelial cancer primary (n = 330)
Observational/registry studies (n = 36)
Pan-tumour basket trials ≥5 cancers (n = 58)
Diagnostic studies (n = 16)
Non-antitumour supportive care (n = 11)
Other ineligible records (n = 58)
Reports sought for retrieval
(n = 1,364)
Reports not retrieved
(n = 0)
Reports assessed for eligibility
(n = 1,364)
Reports excluded: (n = 340)
Intervention confirmed non-pharmacological (n = 142)
Non-bladder/urothelial cancer patients (n = 58)
Insufficient data / unconfirmed eligibility (n = 17)
Lack of data (n = 123)
Included
Registry trials included (n=1,024)
