# Supplementary material for: The clinical landscape of bladder cancer treatment: a brief report on new developments and future perspectives in pharmaceutical interventions
Source: Front Oncol. 2026 Sep 18;16:1747070. doi: 10.3389/fonc.2026.1747070 (PMC13631784; doi:10.3389/fonc.2026.1747070)

Supplementary Figure S2

Current Drug Development Pipeline in Bladder Cancer Therapy

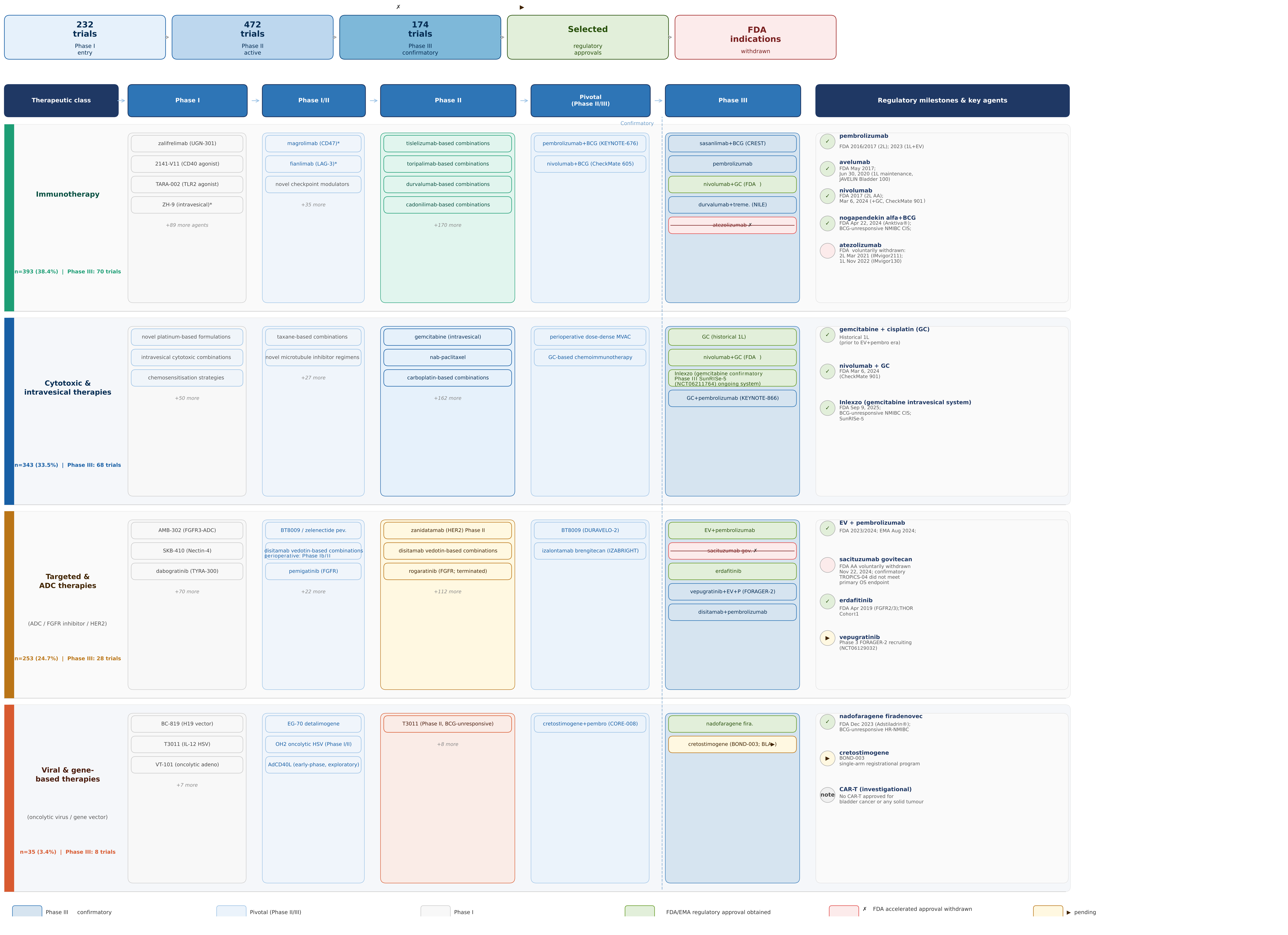

Supplement: Supplementary file 2 [file DataSheet2.pdf]
