# Supplementary material for: The clinical landscape of bladder cancer treatment: a brief report on new developments and future perspectives in pharmaceutical interventions
Source: Front Oncol. 2026 Sep 18;16:1747070. doi: 10.3389/fonc.2026.1747070 (PMC13631784; doi:10.3389/fonc.2026.1747070)
Supplement: Supplementary file 3 [file DataSheet3.pdf]

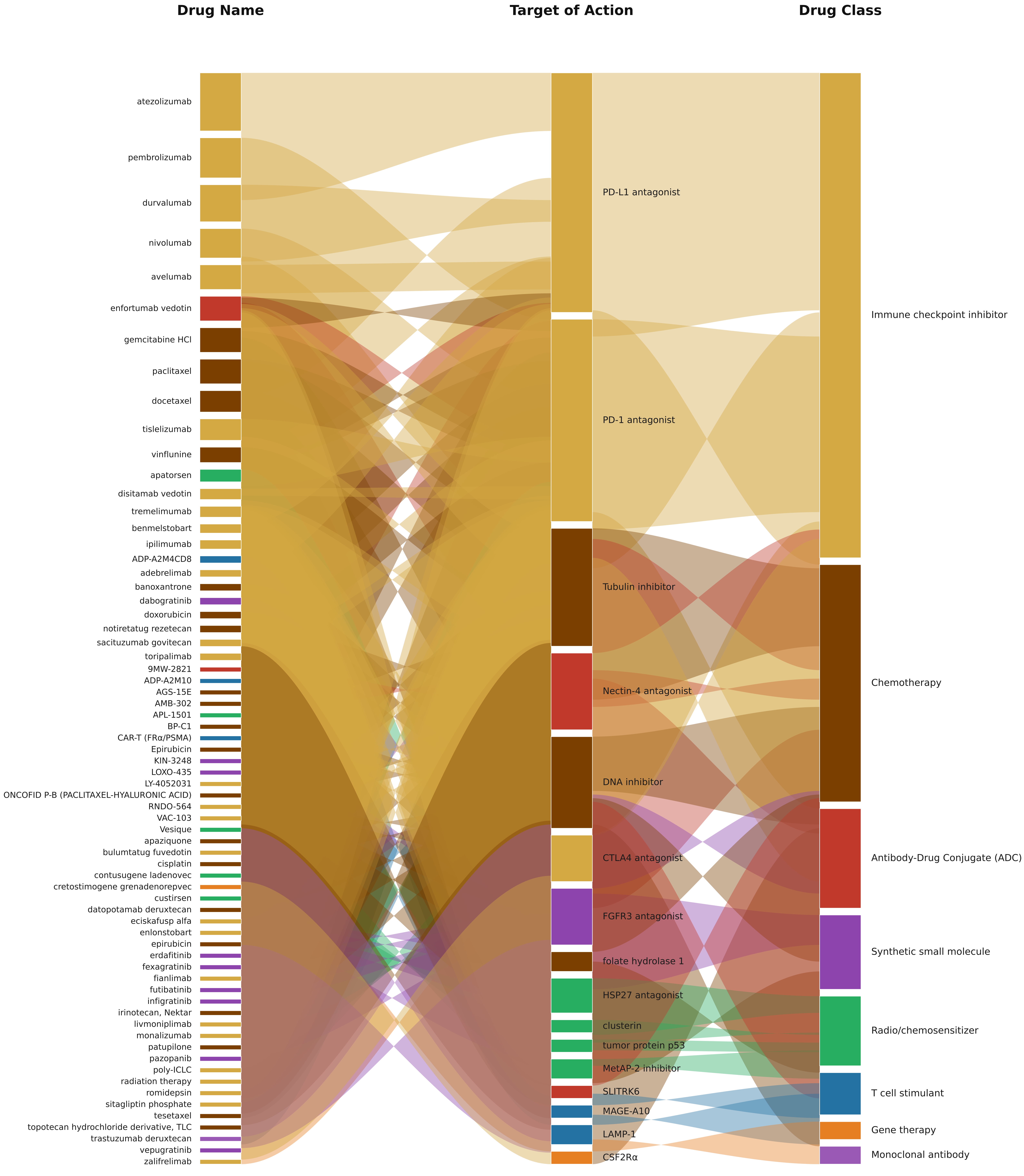

Drug Name
Target of Action
Drug Class
atezolizumab
pembrolizumab
durvalumab
nivolumab
avelumab
enfortumab vedotin
gemcitabine HCl
paclitaxel
docetaxel
tislelizumab
vinflunine
apatorsen
disitamab vedotin
tremelimumab
benmelstobart
ipilimumab
ADP-A2M4CD8
adebrelimab
banoxantrone
dabogratinib
doxorubicin
notiretatug rezetecan
sacituzumab govitecan
toripalimab
9MW-2821
ADP-A2M10
AGS-15E
AMB-302
APL-1501
BP-C1
CAR-T (FRα/PSMA)
Epirubicin
KIN-3248
LOXO-435
LY-4052031
ONCOFID P-B (PACLITAXEL-HYALURONIC ACID)
RNDO-564
VAC-103
Vesique
apaziquone
bulumtatug fuvedotin
cisplatin
contusugene ladenovec
cretostimogene grenadenorepvec
custirsen
datopotamab deruxtecan
eciskafusp alfa
enlonstobart
epirubicin
erdafitinib
fexagratinib
fianlimab
futibatinib
infigratinib
irinotecan, Nektar
livmoniplimab
monalizumab
patupilone
pazopanib
poly-ICLC
radiation therapy
romidepsin
sitagliptin phosphate
tesetaxel
topotecan hydrochloride derivative, TLC
trastuzumab deruxtecan
vepugratinib
zalifrelimab
PD-L1 antagonist
PD-1 antagonist
Tubulin inhibitor
Nectin-4 antagonist
DNA inhibitor
CTLA4 antagonist
FGFR3 antagonist
folate hydrolase 1
HSP27 antagonist
clusterin
tumor protein p53
MetAP-2 inhibitor
SLITRK6
MAGE-A10
LAMP-1
CSF2Rα
Immune checkpoint inhibitor
Chemotherapy
Antibody-Drug Conjugate (ADC)
Synthetic small molecule
Radio/chemosensitizer
T cell stimulant
Gene therapy
Monoclonal antibody
