# Supplementary material for: The clinical landscape of bladder cancer treatment: a brief report on new developments and future perspectives in pharmaceutical interventions
Source: Front Oncol. 2026 Sep 18;16:1747070. doi: 10.3389/fonc.2026.1747070 (PMC13631784; doi:10.3389/fonc.2026.1747070)
Supplement: Supplementary file 4 [file Table3.docx]

**Supplementary Table 3. Distribution of Bladder Cancer Clinical Trials by Therapeutic Category**

| **A. Distribution by Clinical Phase (N = 1,024)** | | | | | | | |
| --- | --- | --- | --- | --- | --- | --- | --- |
| **Therapeutic Category** | **Phase I** | **Phase I/II** | **Phase II** | **Phase II/III** | **Phase III** | **Phase IV** | **Total** |
| **Chemotherapy** | 53  *(15.5%)* | 30  *(8.7%)* | 168  *(49.0%)* | 13  *(3.8%)* | 68  *(19.8%)* | 11  *(3.2%)* | 343  *(100.0%)* |
| **Immunotherapy** | 96  *(24.4%)* | 38  *(9.7%)* | 178  *(45.3%)* | 6  *(1.5%)* | 70  *(17.8%)* | 5  *(1.3%)* | 393  *(100.0%)* |
| **Targeted therapy** | 73  *(28.9%)* | 25  *(9.9%)* | 115  *(45.5%)* | 7  *(2.8%)* | 28  *(11.1%)* | 5  *(2.0%)* | 253  *(100.0%)* |
| **Gene therapy** | 10  *(28.6%)* | 4  *(11.4%)* | 11  *(31.4%)* | 1  *(2.9%)* | 8  *(22.9%)* | 1  *(2.9%)* | 35  *(100.0%)* |
| **Total** | **232**  *(22.7%)* | **97**  *(9.5%)* | **472**  *(46.1%)* | **27**  *(2.6%)* | **174**  *(17.0%)* | **22**  *(2.1%)* | **1024**  *(100.0%)* |

| **B. Distribution by Trial Status (N = 1,024)** | | | | | | |
| --- | --- | --- | --- | --- | --- | --- |
| **Therapeutic Category** | **Open** | **Completed** | **Terminated** | **Closed** | **Planned** | **Total** |
| **Chemotherapy** | 87  *(25.4%)* | 156  *(45.5%)* | 48  *(14.0%)* | 30  *(8.7%)* | 22  *(6.4%)* | 343  *(100.0%)* |
| **Immunotherapy** | 181  *(46.1%)* | 111  *(28.2%)* | 47  *(12.0%)* | 42  *(10.7%)* | 12  *(3.1%)* | 393  *(100.0%)* |
| **Targeted therapy** | 93  *(36.8%)* | 79  *(31.2%)* | 39  *(15.4%)* | 15  *(5.9%)* | 27  *(10.7%)* | 253  *(100.0%)* |
| **Gene therapy** | 18  *(51.4%)* | 9  *(25.7%)* | 6  *(17.1%)* | 0  *(0.0%)* | 2  *(5.7%)* | 35  *(100.0%)* |
| **Total** | **379**  *(37.0%)* | **355**  *(34.7%)* | **140**  *(13.7%)* | **87**  *(8.5%)* | **63**  *(6.2%)* | **1024**  *(100.0%)* |
