# Supplementary material for: The clinical landscape of bladder cancer treatment: a brief report on new developments and future perspectives in pharmaceutical interventions
Source: Front Oncol. 2026 Sep 18;16:1747070. doi: 10.3389/fonc.2026.1747070 (PMC13631784; doi:10.3389/fonc.2026.1747070)
Supplement: Supplementary file 5 [file Table1.doc]

Supplementary Table S1. Detailed Search Strategies for Three Clinical Trial Databases

| **#** | **Database** | **Search Field** | **Search String / Criteria** | **Additional Filters** |
| --- | --- | --- | --- | --- |
| **Database 1 — Trialtrove (Citeline Clinical Intelligence) \| Search date: December 31, 2025 \| URL: https://trialtrove.citeline.com** | | | | |
| 1.1 |  | **Drug Disease** | “Bladder cancer” | AND logic (combined with 1.2–1.4)  All countries; all languages |
| 1.2 |  | **Drug Disease Group** | “Oncology” | AND |
| 1.3 |  | **MeSH Term ID** | D008495  (Urinary Bladder Neoplasms)  D001749  (Urinary Bladder Diseases) | AND  Verified: NLM MeSH Browser (meshb.nlm.nih.gov). D008495 = primary descriptor; D001749 = broader parent term for sensitivity |
| 1.4 |  | **Trial Type** | [Filter]: Interventional | Excludes observational registries,  expanded access entries |
| **Database 2 — ClinicalTrials.gov (U.S. National Library of Medicine) \| Search date: December 31, 2025 \| URL: https://clinicaltrials.gov** | | | | |
| 2.1 |  | **Condition / Disease (free-text)** | “bladder cancer”  OR “urinary bladder neoplasms”  OR “bladder carcinoma”  OR “urothelial carcinoma”  OR “bladder urothelial carcinoma”  OR “transitional cell carcinoma of the bladder”  OR “vesical carcinoma”  OR “muscle invasive bladder cancer”  OR “non-muscle invasive bladder cancer” | Boolean: OR within field  AND with 2.2  Study Type = Interventional  Age = Adult (≥18 years)  No date range restriction  All languages  All phases |
| 2.2 |  | **Intervention Type (filter)** | [Filter]: Drug \| Biological \| Genetic | AND with 2.1  pharmacological agents only |
| 2.3 |  | **Logic** | (Condition Terms) AND (Intervention Type Filter) | — |
| **Database 3 — WHO ICTRP (International Clinical Trials Registry Platform) \| Search date: December 31, 2025 \| URL: https://trialsearch.who.int** | | | | |
| 3.1 |  | **Condition (free-text)** | “bladder cancer”  OR “bladder carcinoma”  OR “urothelial carcinoma”  OR “bladder urothelial carcinoma”  OR “transitional cell carcinoma of the bladder”  OR “vesical carcinoma”  OR “muscle invasive bladder cancer”  OR “non-muscle invasive bladder cancer” | Boolean: OR within field  AND with 3.2  Recruitment status = All  No date range restriction  All languages  All 17 contributing registries |
| 3.2 |  | **Intervention Type (filter)** | [Filter]: Drug \| Biological \| Genetic | AND with 3.1  Same restriction as ClinicalTrials.gov (2.2) for cross-database consistency |
| 3.3 |  | **Logic** | (Condition Terms) AND (Intervention Type Filter) | — |
